# Symptoms of depression, sadness and sense of coherence (coping) among cognitively intact older people with cancer living in nursing homes—a mixed-methods study

**DOI:** 10.7717/peerj.2096

**Published:** 2016-06-09

**Authors:** Jorunn Drageset, Geir Egil Eide, Solveig Hauge

**Affiliations:** 1 Faculity of Health and Social Science, Bergen University College, Bergen, Norway; 2 Department of Global Public Health and Primary Care, Faculty of Medicine and Dentistry, University of Bergen, Bergen, Norway; 3 Centre for Clinical Research, Western Norway Health Region Authority, Bergen, Norway; 4 Faculty of Health and Social Studies and Centre for Caring Research–Southern Norway, Unversity College of Southeast, Porsgrunn, Norway

**Keywords:** Depression, Coping, Sadness, Nursing home, Cancer, Mix-methods

## Abstract

**Background:** Symptoms of depression are often reported among patients with a cancer diagnosis. Strong sense of coherence (SOC) is shown to be associated with less depression in the general older population and among nursing homes (NH) residents in particular. Knowledge about mixed-methods perspectives that examine symptoms of depression and SOC among cognitively intact NH residents with cancer is scarce.

**Aim:** To investigate symptoms of depression and SOC among NH residents who are cognitively intact and have cancer.

**Methods:** We used a quantitatively driven mixed-methods design with sequential supplementary qualitative components. We facilitated the collection of quantitative survey data of 60 NH residents (≥ 65 years) with cancer using the Geriatric Depression Scale (GDS) and SOC scale. The supplementary psychosocial component comprised qualitative research interviews about experiences related to depression with nine respondents from the same cohort.

**Results:** In fully adjusted multiple regression analysis of the sociodemographic variables, the GDS was significantly correlated with SOC (*P* < 0.001). The experience of sadness was identified by the following theme: sadness. Coping with the experience of symptoms of depression was dominated by coping with sadness.

**Conclusion:** More than half the NH residents reported symptoms of depression, and the SOC was associated with reduced symptoms. A mixed-methods design contributed to nuanced and detailed information about the meaning of depression, and the supplementary component informs and supports the core component. To improve the situation of NH residents with cancer, more attention should be paid to the residents’ experience of symptoms of depression and their SOC.

## Introduction

Nursing home (NH) residents with cancer are given little attention (Duncan, Forbes-Thompson & Bott, 2008). Since the incidence of cancer increases with age, more people will be diagnosed with cancer in the years ahead as the number of older people is growing (Bourbonniere & Van Cleave, 2006; International Agency for Research on Cancer, 2012). Many of these older people will live in NH (Clement, Bradley & Lin, 2009).

At admission to a NH, 10–26% of the residents have a diagnosis of cancer (Drageset, Eide & Ranhoff, 2012; Rodin, 2008), and some develop cancer while living in the NH (Clement, Bradley & Lin, 2009). Because of their actual condition and treatment, many of these NH residents with cancer experience distressing symptoms such as fatigue, anorexia, nausea, vomiting and pain (Drageset et al., 2014; Pimentel et al., 2015) and have unstable health (Bourbonniere & Van Cleave, 2006; Buchanan et al., 2005). These symptoms cause great concern to people with cancer and their families, increase stress, anxiety and the experience of depression and reduce health-related quality of life (Barkin, Barkin & Barkin, 2005). Symptoms of depression are described as the presence of sad, empty or irritable mood accompanied by somatic and cognitive changes that significantly affect these people’s capacity to function. What differs among them are issues of duration, timing and presumed cause (American Psychiatric Association, 2013).

Symptoms of depression have been reported to be frequent in NH populations (Drageset, Eide & Ranhoff, 2011; Haugan, 2014; Iden et al., 2014; Johnson et al., 2005), and much more frequent (*n* = 72, 32%) than the respective diagnoses in the medical records (depression diagnosis, *n* = 40, 18%) (Drageset, Eide & Ranhoff, 2013a). Among residents with cancer, the prevalence of diagnosed depression has been shown to be 26% (Buchanan et al., 2005), and symptoms of depression are often reported (Matulonis, 2004). Symptoms of depression have been shown to be associated with death regardless of cancer among cognitively intact NH residents (Drageset, Eide & Ranhoff, 2013b), and the coexistence of cancer and symptoms of depression has been shown to be associated with a significantly increased risk of death (Onitilo, Nietert & Egede, 2006). In addition to having a diagnosis of cancer, the residents often moved to NH as a result of illness and several losses such as loss of physical functioning, significant others, home, autonomy and privacy. Consequently, grief and symptoms of depression might occur that deeply affect NH residents’ lives and contribute to the experience of sadness and, in turn, depression. Sadness seems to be part of the human condition (Horwitz & Wakefield, 2007). The prevalence and intensity vary between individuals and cultures, and depression symptoms are expressed in various ways and associated with different experiences. Normal sadness varies according to each person’s life situation and may decline when the situation changes. Sadness can be a normal emotional reaction but is also a core symptom of depression (Horwitz & Wakefield, 2007). Knowledge on how residents cope is therefore vital to enable NH residents to cope with their different challenges such as sadness and depression.

Meeting these challenges requires understanding how NH residents with cancer define, perceive and cope with the experience of symptoms of depression. In this context, exploring significant factors, such as sense of coherence (SOC), would be of interest. Antonovsky (1987) introduced the concept of SOC to explain human coping resources. People who possess a strong SOC are able to comprehend their present life situation, find it meaningful and maintain high quality of life despite declining health (Antonovsky, 1987). The assumption is that strong SOC is associated with resources to cope with various kinds of stressful life events or situations. SOC is postulated to have three components: comprehensibility (that stimuli from one external and internal environment are structured, explicable and predictable) manageability (the extent to which resources are available to a person to meet the demands posed by these stimuli) and meaningfulness (the extent to which these demands are challenges worthy of investment and engagement). Further, SOC indicates an individual’s general resistance resources and the ability to make use of these resources. General resistance resources represent integral biological, material and psychosocial resources supporting individuals in perceiving their lives as consistent, structured and understandable. Previous research indicates that a strong SOC is associated with less depression in the general older population (Bjørkløf et al., 2013; Eriksson & Lindström, 2006) and among NH populations in particular (Drageset, Espehaug & Kirkevold, 2012).

Knowledge and understanding about the relationships between symptoms of depression, how people cope and the residents’ experiences may improve the care of NH residents with cancer. This requires extending the perspectives beyond what one specific research approach can give.

## Aim

This study investigated the SOC and depression among cognitively intact NH residents with cancer and their experience with depression and coping. The specific research questions were as follows. How is depression associated with sociodemographic variables, comorbid illnesses and SOC? How do NH residents with cancer describe experiences of depression and coping with depression?

## Methods

### Design and population

We used a quantitatively driven mixed-methods design with qualitative sequential components (Morse & Niehaus, 2009). The theoretical drive, or the deductive direction of a research project, guides the quantitative methodological core (Morse et al., 2006) and enables the investigation of symptoms of depression and coping quantitatively for testing and refuting previously developed concepts (Morse & Niehaus, 2009). These methods differ from other approaches because the quantitative core component is guiding the study, and may stand alone, and the supplementary qualitative component is used to expand certain details of the findings to indicate the validity of the core findings (Morse & Niehaus, 2009).

The quantitative core component initially comprised 60 respondents with cancer, and we facilitated the collection of the survey data using the Geriatric Depression Scale (GDS) and SOC questionnaire. The supplementary component comprised qualitative research interviews about life experiences related to symptoms of depression and how to cope with these symptoms from the same respondents. The supplementary component enabled us to explore what the experience meant to the respondents, to support the quantitative results.

**Figure 1:**
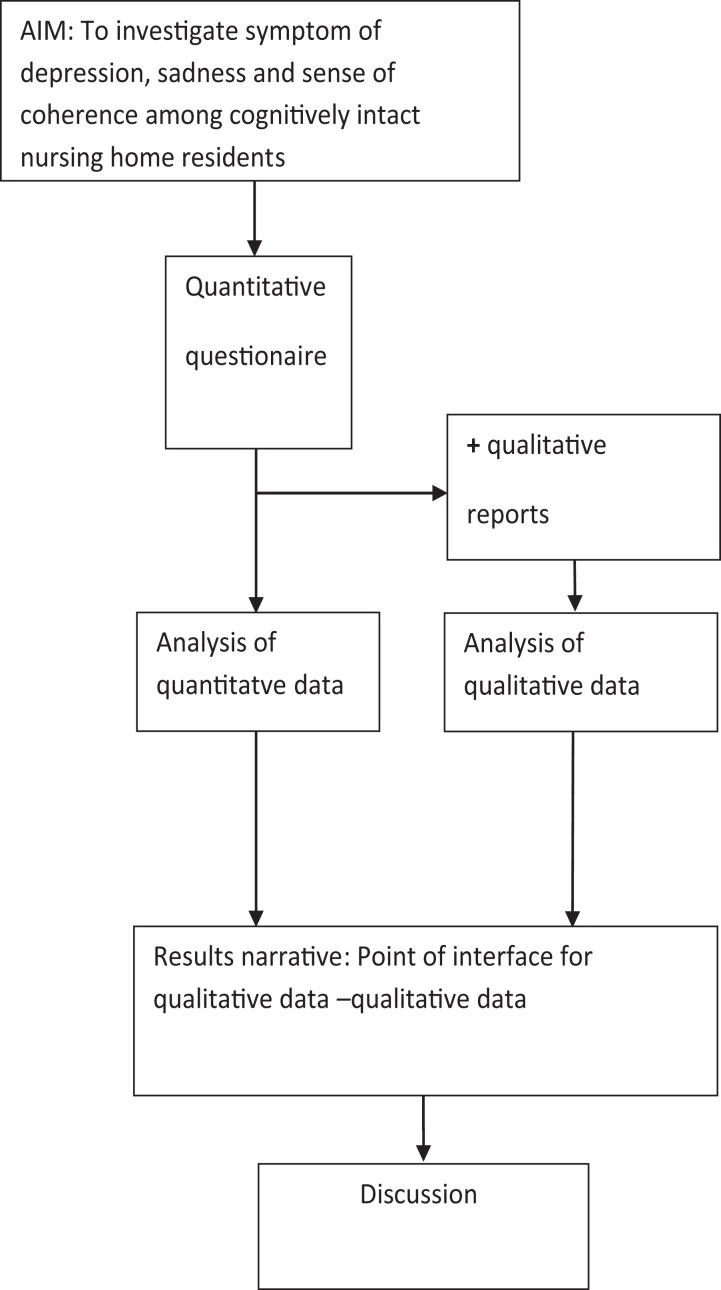
Schematic overview of QUAN-qual mixed-method design.

Once we analyzed the quantitative core components and completed the supplementary qualitative components, we described the findings for the core component findings. We then integrated the final descriptions from qualitative components, and these constitute a result narrative on which the discussion is based (Fig. 1).

### Sampling and recruitment

This study is part of a study (*n* = 227, 167 without cancer and 60 with cancer) conducted in 2004–2005 (Drageset et al., 2009) with follow-up until 2011. At the end of follow-up, 19 NH residents were still alive, and of these, the qualitative part of the study included nine residents with cancer. The inclusion criteria were 65 years and older, cognitively intact, capable of conversing and residing in the NH for at least 6 months. Cognitively intact was defined as having a Clinical Dementia Rating (CDR) ≤ 0.5 (Hughes et al., 1982). The CDR was developed as a staging instrument for dementia and is scored as no (0), questionable (0.5), mild (1), moderate (2) and severe (3) dementia, and the overall level of dementia is derived by using a standard algorithm (Morris, 1993). As part of the recruitment process, trained nurses who had observed the residents for at least four weeks assessed CDR and were instructed to base their CDR scoring on mental functioning and not to include physical frailty. The CDR has shown high interrater reliability for physicians and other health professionals (McCulla et al., 1989). Exclusion criteria were: lived less than six months in a NH, CDR score > 0.5 and residents who could not converse with the researcher as determined by a doctor or nurse assessing general health status. A primary care nurse invited the residents to participate.

#### Ethical approval

The data were collected during 2009–2010. Informed consent was obtained. The Western Norway Regional Committee for Medical and Health Research Ethics and the Norwegian Social Science Data Services approved the study (REK. Vest number. 62.03/2009/1550).

### Data collection

#### Quantitative data

We facilitated the collection of survey data from 60 residents with a cancer diagnosis in the respondent’s room or at another appropriate place in the NH. The principal investigator (JD) interviewed the residents, including reading the questions to the participants, circling the indicated answers and recording demographic information. This was required, since many residents have problems holding a pen and have impaired vision.

#### Assessment instruments

We collected sociodemographic variables such as age, sex, marital status, educational level and information about comorbidity from records. We scored comorbidity using the Functional Comorbidity Index (FCI), a clinically based measure (Groll et al., 2005). This Index includes 18 diagnoses scored “yes = 1” and “no = 0”. A maximum score of 18 indicates the highest number of comorbid illnesses.

### Geriatric Depression Scale

We collected survey data on the symptoms of depression by using the GDS (Yesavage et al., 1982). The GDS was originally developed as a 30-item instrument but was shortened to 15 of the 30 original items. The short version of the GDS has been found to be reliable and valid among older people in different settings (Lesher & Berryhill, 1994; Sheikh & Yesavage, 1986), including NH (Jongenelis et al., 2005; McGivney, Mulvihill & Taylor, 1994) and has been recommended for use in NH populations (McGivney, Mulvihill & Taylor, 1994; Smalbrugge et al., 2008). The GDS was developed to be self-administered, but among frail NH residents it is frequently administered in an interview (Jongenelis et al., 2007) on a scale from 0 (no depression) to 15.

### Sense of Coherence questionnaire

Based on his salutogenic health theory involving SOC, Antonovsky (1987) developed the original 29-item Orientation to Life Questionnaire (OLQ-29) for measuring SOC. Later, the 13-item version of this questionnaire was developed; this study used the Norwegian version of the OLQ-13. The 13 items are rated on a 7-point scale with two anchoring verbal responses: for example, “very seldom or never” and “very often.” The total sum ranges from 13–91, and a higher score indicates stronger SOC (Antonovsky, 1987).

#### Qualitative data

We collected data through individual qualitative research interviews. We developed a semistructured interview guide based on our previous research findings (Drageset, Eide & Ranhoff, 2013a, Drageset, Eide & Ranhoff, 2013b).

We asked informants about their experience of depression and coping with depression. We performed interviews in each resident’s room, and they lasted 45–90 min. The interviewer (the first author) conducted the interviews as conversations in which participants were prompted to describe their experience regarding the questions in the interview guide. The interviewer asked spontaneous follow-up questions based on the respondents’ answers to the questions. We taped the interviews and transcribed them verbatim.

### Data analysis

#### Statistical analysis

We calculated descriptive statistics for the demographic variables, comorbidity, symptoms of depression and the SOC. We used linear regression analysis to study whether GDS depends on the SOC. We adjusted for sex, age, marital status, education and Groll’s FCI. The results are given as estimated regression coefficients (*b*), 95% confidence intervals (CI) and determination coefficient (*R*^2^). We included SOC and comorbidity in the model as continuous covariates but coded the rest of the variables as categorical. We examined the residuals for deviations from the usual assumptions for standard linear regression. We used IBM SPSS Statistics (Version 21.0) for all statistical analysis. We defined *P* < 0.05 as statistical significance.

#### Qualitative data analysis

We analyzed the interview data by elements of qualitative content analysis (Graneheim & Lundman, 2004; Kvale & Brinkmann, 2009). The analytical process occurred in six stages: 1) transcription of the interview; 2) open independent reading of all material to gain an overall impression of the text; 3) identification of meaning units; 4) categorization; 5) abstracting eight subthemes and two themes; and 6) reflection and discussion.

## Results

### Respondents’ characteristics

Of the 60 respondents, 39 (65%) were women (Table 1). The mean age was 85.3 years (SD 6.7). The mean number of comorbid illnesses was 2.2 (median 2.0, SD 1.3, range 0–6).

**Table 1:** Personal characteristics of the 60 respondents.

	With cancer (n = 60)
Characteristic	No. (%)
**Sex**	
Men	21 (35.0)
Women	39 (65.0)
**Age (years)**	
65–74	3 (5.0)
75–84	24 (40.0)
85–94	28 (46.7)
≥ 95	5 (8.3)
**Marital status**	
Married or cohabiting	18 (30.0)
Unmarried	8 (13.3)
Divorced	4 (6.7)
Widowed	30 (60.1)
**Education**	
Primary school	22 (36.7)
< 3 years after primary school	31 (51.7)
≥ 3 years after primary school	7 (11.7)
**Groll’s index (FCI^a^)**	
FC ≥ 1	53 (88.3)
FCI = 0	7 (11.7)
**GDS score^b^**	
1–4	37 (45.0)
5–15	33 (55.0)

**Notes:**

a FCI: Functional Comorbidity Index (0–18).

b GDS score 1–4; 5–15.

### Quantitative findings

A total of 55% of the participants reported symptoms of depression. The mean (SD) GDS score was 5.8. GDS was significantly negatively associated with SOC in simple linear regression (*b* = −0.157, 95% CI: −0.185 to −0.128, *P* < 0.001, *R*^2^ = 0.34) (Table 2). This association did not change substantially after adjustment for sex, age group, marital status, education and Groll’s FCI (*b* = −0.170, 95% CI: −0.232 to −0.108, *P* < 0.001, adjusted *R*^2^ = 0.40). Although the residuals were not strictly normally distributed, they showed a sufficiently symmetrical shape to make the statistical inference valid.

**Table 2:** Lineær regression analysis of GDS for 60 cognitive intact NH residents with cancer.

	Unadjusted^a^			Fully adjusted^b^		
	B	95% CI^c^	*P*	B	95% CI^c^	*P*
**Sex**						
Men	−0.612	(−2.57, 1.35)	0.534	−6.37?	(−2.59, 1.35)	
Women	Reference			Reference		
**Age (years)**			0.316			
65–74	2.733	(−3.46, 0.86)		−6.740	(−6.73, 2.61)	
75–84	1,942	(−1.58, 5.46)			(−0.82, 5.15)	
85–94	0.364	(−3.11, 3.83)			(−2.64, 3.04)	
≥ 95	Reference			Reference		
**Education**			0.200			
Primary school	2.753	(0.33, 5.84)		0.749	(−1.95, 3.45)	
< 3 years after primary school	2.410	(0.57, 5.39)		1.206	(−1.27, 3.68)	
≥ 3 years after primary school	Reference			Reference		
**Marital status**			0.527			
Married or cohabiting	−1.300	(−3.46, 0.86)		−1.725	(−3.79, 0.35)	
Unmarried	0.408	(−2.47, 3.29)				
Divorced	0.783	(−3.07, 4.64)				
Widowed	Reference					
**Groll’s index**	−0.531	(−1.24, 0.19)	0.143	−0.338	(−0.93, 0.25)	
**SOC**	−0.157	(−0.19, 0.13)	< 0.001	0.170	(0.23, 0.11)	< 0.001

**Notes:**

a Unadjusted for sex, age group, marital status, educational level, comorbidity.

b Fully adjusted for sex, age group, marital status, educational level, comorbidity.

c CI: Confidence interval.

### Qualitative findings

#### Experience of sadness

Some participants described a strong feeling of sadness. The feelings of sadness were outlined as a combination of grief, of experiencing different types of loss and a feeling of worthlessness (Table 3).

**Table 3:** Overview of categories, subthemes and themes in qualitative analyses.

Categories	Sub-themes	Themes
Feel sorrow	Grief	
Inner pain		
Yearning		
Loss of integrity		Sadness
Loss of significant others	Feelings of loss	
Loss of health		
Overlooked by caregiver		
No one cares	Worthless	
Be alone and isolated		
Family	Connectedness	
Friends		
Respect	Confirm their identity	
Privacy		Coping with sadness
Notify	Integrated	
Determination		
Practical help		
Be self-reliant	Independent	
Aids		
Crafts	Being engaged	
Being outdoors		
Using the senses (*Radio and TV*)		

Grief was described as inner pain: “I cry many times, particular in the evenings” (PT001, female, chronic illnesses). The participants felt sad when thinking of their situation as a whole. They also longed back to earlier days when they were independent of the help of other people and lived in their own homes.

Sadness was further connected to all the losses the participants described. They felt that they had lost their health, significant others and their own integrity. “Earlier I could help myself, but not anymore” (PT002, female, chronic illnesses). “I have lost my strength, and I cannot read or watch TV anymore” (PT003, female, chronic illnesses). They also felt that they had lost their family when moving to the NH. “I feel sad when thinking of my family” (PT004, male, chronic illnesses). Further, the way they describe sadness may indicate that sadness made them feel worthless. This was especially connected to how the caregiver acted. “A lot of things hurt me. I feel overlooked by many of the caregivers. I feel like nobody” (PT005, female, chronic illnesses). The feeling of being worthless was also connected to such experiences as: “I feel tired of being here. It feels like I’m trapped because I cannot go out, the caregivers have no time …” (PT006, female, chronic illnesses). “The days are so long–I get tired in a way … do not do anything … everything is just nonsense. I am not able to do the things I want to do …” (PT007, male, chronic illnesses).

#### Coping with sadness

The residents also described strategies to counteract sadness such as connectedness, confirmation of identity, independence and engagement (Table 3).

Connectedness was usually related to their closest family. “I have a lot of relatives, and some of them are very important. Especially my children” (PT008, female, chronic illnesses). But some residents still had friends with whom they felt connected. For them, it was important to be able to talk to their friends, either face to face or by telephone.

Another important aspect that contributed to the residents’ ability to cope with sadness was experiences that confirmed their identity. Experiences of respect, for instance in dialog with the caretakers, were very important, but arrangements that are more practical were also crucial. For instance: “I have my own room in the nursing home. I don’t have to share a room with other residents” (PT009, male, chronic illnesses). Further, some residents talked about the importance of speaking up about their wishes. “It is important that I say what I think about things” (PT004, female, chronic illnesses).

A certain feeling of independence was important for the residents. Even if they all needed considerable help with everyday activities, some were very eager to do whatever they were able to: for instance, during morning care. “I like to help myself with some practical things, such as putting on some of my clothes. I can manage some things using practical aids” (PT009, male, chronic illnesses). Another resident explained it like this: “It is important for me to be persistent and really try to be as independent as possible” (PT009, male, chronic illnesses).

Being engaged was another important dimension for coping with sadness. The residents described such activities as listening to the radio and watching TV, and some were able to keep up with some handicrafts, such as knitting. “It makes me happy than I can knit” (PT001, female, chronic illnesses). Being able to get outdoors was also very welcome. “You have to find something to do during the day” (PT008, female, chronic illnesses).

## Discussion

### Results narrative

We combined the findings of the quantitative and the qualitative data analysis to produce the results narrative, enabling deeper critical interpretation. Both datasets showed experiences of symptoms of depression and sadness. Findings from both the quantitative data and the qualitative findings highlight that coping was essential for the experience of symptoms of depression and sadness. The quantitative data showed that SOC is important for reducing symptoms of depression. The qualitative data provided many descriptions of the experiences of sadness and how to cope with these experiences. The combined findings call for some improvements in care among NH residents concerning how the residents cope with the experiences of sadness.

This study showed that 55% of cognitively intact NH residents with cancer reported symptoms of depression. In addition, the supplementary components describe symptoms of depression as experiencing sadness.

Further, this study showed that, as the SOC score increased, the number of symptoms reported decreased among cognitively intact NH residents with a diagnosis of cancer. Symptoms of depression have been reported in several studies among NH residents with cancer (Buchanan et al., 2005; Johnson et al., 2005; Matulonis, 2004).

Qualitative data supported the symptoms of depression from the quantitative data, and the qualitative data on sadness were described as a combination of grief, of experiencing different losses and a feeling of worthlessness.

Our findings on the feelings of grief suggest that dependence on other people could be one of the causes. This findings are in accordance with a qualitative study of NH residents that described sadness as functional ability and dependence on long-term care (Iden, Ruths & Hjørleifsson, 2015) and a quantitative study reporting that symptoms of depression were connected to dependence in the activities of daily living among NH residents (Drageset, Eide & Ranhoff, 2011). In addition, the participants described the various losses as loss of their health, significant others and their own integrity. Having a cancer diagnosis may represent an additional burden that may create losses such as limited functioning and declining health beyond the normal aging processes (Buchanan et al., 2005). The residents’ experiences regarding loss of integrity are similar to the experiences of other NH residents (Choi, Ransom & Wyllie, 2008; Iden, Ruths & Hjørleifsson, 2015) that describe symptoms of depression as loss of family and friends and lack of privacy and autonomy.

Our findings regarding the association between symptoms of depression and SOC indicate that higher SOC contributes to fewer reported symptoms of depression. This is in accordance with the findings of Eriksson & Lindström (2006) and Drageset, Espehaug & Kirkevold (2012), which showed a positive relationship between SOC and health. Higher SOC will make the situation for the residents with cancer more compressible, manageable and meaningful. The residents’ experienced connectedness with close family can be seen in the light of Antonovsky’s (1987) theory as a way of supporting their comprehensibility. The communication with close family may give a sense of control and a feeling of safety and security by having a person that gives them support when they need it. Confirming their identity could be understood as the component of manageability. According to Antonovsky’s (1987) theory, manageability is described as the extent to which one perceives that one has the resources necessary to meet the demands posed by various stimuli. Resources at their disposal can include being able to communicate with nurses and the feeling of being as independent as possible. An interview study with NH residents in the Netherlands (Oosterveld-Vlug et al., 2013) indicated that the feeling of having control of one’s life and the feeling of being a worthwhile person were important in maintaining dignity. These were outlined as physical improvement and being socially involved with NH personnel, other residents and relatives. In addition, in a qualitative study in Norway among people with cancer, Kvåle & Synnes (2013) found that doctors and nurses can be a vital resource at the disposal of the people with cancer as they need it.

The residents tried to find meaning in daily life, including activities during the day to remain engaged. In Antonovsky’s (1987) view, meaningfulness seems to be the most crucial component in the SOC as a whole. The residents meet the challenges regarding sadness and seek meaningfulness in activity. According to the theory, a person with a high degree of meaningfulness will be determined to seek meaning despite the experiences. Other studies have highlighted that meaningfulness is essential for the quality of life among NH residents and that the residents’ own attitudes towards their life situation were an important factor for thriving (Bergland & Kirkevold, 2006; Hjaltadóttir & Gústafsdóttir, 2007).

We found an association between SOC and GDS. This may indicate that some residents select an appropriate coping strategy to deal with this stressor. This enables them to achieve more control of the situation and thus preserve their identity and feel safe. NH personnel, other NH residents and family members seemed to be vital resources available when required. General resistance resources are important for promoting and maintaining meaningfulness, comprehensibility and manageability: the components that comprise SOC.

## Methodological Considerations

The design with mixed methods enabled us to clarify more widely various aspects of depression (Morse & Niehaus, 2009). Using both qualitative and quantitative methods provides more holistic (complete) answers to our research questions. The supplementary qualitative data analysis informed and supported the core component: quantitative data. Thus, the mixed-methods design validated and extended our findings in accordance with the rules inherent in each paradigm (Morse et al., 2006).

To enhance credibility, experienced qualitative researchers transformed the transcribed qualitative data from meaning units into themes and discussed how to interpret their textual meaning. However, this study included only residents with cancer, so the data do not offer insight into whether the answers concerning symptoms of depression resulted from cancer, NH residence or old age in general.

The strengths of this study include all participants being interviewed face to face, thus preventing possible misunderstandings in meaning, which adds to the study’s validity. The residents reported their narratives about symptoms of depression and coping themselves, which probably strengthens the validity of the meanings in the text. Further, this study followed a cohort (*n* = 60) of frail cognitively intact NH residents from 2004–2005 to 2011, and nine NH residents with cancer were still alive at the end of follow-up. The study sample of 60 subjects is representative for the cognitively intact NH residents with cancer for this period in the region. Based on this, we found meaningful qualitative findings that extended and supported the quantitative results.

## Conclusion

More than half the NH residents reported symptoms of depression, and the SOC was associated with reduced symptoms. Our mixed-methods design provided detailed and nuanced information on the meaning of depression, and the supplementary component informed and supported the core component. Improving the situation of NH residents with cancer requires paying more attention to the residents’ experience of symptoms of depression and their SOC.

## Supplemental Information

10.7717/peerj.2096/supp-1 Supplemental Information 1 COREC checklist.

## Additional Information and Declarations

### Data Deposition

The following information was supplied regarding data availability: According to the Norwegian ACT 2008-06-20 no. 44: Act on medical and health research (the Health Research Act) regulated in the paragraph 7 Duty of confidentiality and 13 Main rule on consent, we cannot make the raw data available.
